# Supplementary material for: Mapping HIV prevalence in Nigeria using small area estimates to develop a targeted HIV intervention strategy
Source: PLoS One. 2022 Jun 8;17(6):e0268892. doi: 10.1371/journal.pone.0268892 (PMC9176772; doi:10.1371/journal.pone.0268892)
Supplement: Attachment — Submitted filename: PONE-D-21-31941 Comments to Authors.docx [file pone.0268892.s001.docx]

**COMMENTS TO AUTHORS**

**REVIEWER 1**

Great efforts put in to utilized population-based HIV survey for evidence or targeted interventions to prevent new HIV infection and HIV control in a resource-limited nation is good innovation.

The STATISTICAL METHODS is robust and results unambiguous for policy guidance in HIV/AIDS programming. However, there are many claims that ensemble models used in your statistical analysis could be shrouded with generalization errors, how did you overcome overfitting models in your analysis?

Data access was adequate and useful in buttressing the credibility of the manuscript.

**REVIEWER 2**

The strength of the study lies on the used household surveys and GPS to analyze data for HIV prevalence, ART and VLS.

This is a better model than the State based approaches used presently in the country for policy making. However, the challenge is that the use of these databases have underlying assumptions, as stated by the authors as limitations to the study, use of household data may not correctly capture high risk groups, vulnerable populations and marginalized communities. Also, the use of GPS may incorrectly capture coordinates for all the locations. These limitations will hamper the reliability of the results.

Suggestion: There are other software used by medical geographers and geospatial analysts that could be used alongside the data used in this study to further enhance the accuracy of the results and also eliminate the bias further. It is not out of place if the authors engage these specialists in order to further improve the quality of the work.

Specific suggestions: Replace "prevalence of ART" with "ART coverage"
Replace "prevalence of VL suppression" with "virus suppression rate"
