# Supplementary material for: Mapping HIV prevalence in Nigeria using small area estimates to develop a targeted HIV intervention strategy
Source: PLoS One. 2022 Jun 8;17(6):e0268892. doi: 10.1371/journal.pone.0268892 (PMC9176772; doi:10.1371/journal.pone.0268892)
Supplement: Attachment — Submitted filename: Response to Reviewers.docx [file pone.0268892.s002.docx]

**Journal Requirements:**

When submitting your revision, we need you to address these additional requirements.

1. Please ensure that your manuscript meets PLOS ONE's style requirements, including those for file naming. The PLOS ONE style templates can be found at 
<https://journals.plos.org/plosone/s/file?id=wjVg/PLOSOne_formatting_sample_main_body.pdf> and 
<https://journals.plos.org/plosone/s/file?id=ba62/PLOSOne_formatting_sample_title_authors_affiliations.pdf>

Response: Files renamed and formatting corrected where needed.

2. Please update your submission to use the PLOS LaTeX template. The template and more information on our requirements for LaTeX submissions can be found at <http://journals.plos.org/plosone/s/latex>.

Response: Files updated to reflect the LaTeX template.

3. We note that Figure(s) 1, 2 and 3 in your submission contain [map/satellite] images which may be copyrighted. All PLOS content is published under the Creative Commons Attribution License (CC BY 4.0), which means that the manuscript, images, and Supporting Information files will be freely available online, and any third party is permitted to access, download, copy, distribute, and use these materials in any way, even commercially, with proper attribution. For these reasons, we cannot publish previously copyrighted maps or satellite images created using proprietary data, such as Google software (Google Maps, Street View, and Earth). For more information, see our copyright guidelines: <http://journals.plos.org/plosone/s/licenses-and-copyright>.
 
We require you to either (1) present written permission from the copyright holder to publish these figures specifically under the CC BY 4.0 license, or (2) remove the figures from your submission:
 
1. You may seek permission from the original copyright holder of Figure(s) 1, 2 and 3  to publish the content specifically under the CC BY 4.0 license.  
 
We recommend that you contact the original copyright holder with the Content Permission Form (<http://journals.plos.org/plosone/s/file?id=7c09/content-permission-form.pdf>) and the following text:
“I request permission for the open-access journal PLOS ONE to publish XXX under the Creative Commons Attribution License (CCAL) CC BY 4.0 (<http://creativecommons.org/licenses/by/4.0/>). Please be aware that this license allows unrestricted use and distribution, even commercially, by third parties. Please reply and provide explicit written permission to publish XXX under a CC BY license and complete the attached form.”
 
Please upload the completed Content Permission Form or other proof of granted permissions as an "Other" file with your submission.
 
In the figure caption of the copyrighted figure, please include the following text: “Reprinted from [ref] under a CC BY license, with permission from [name of publisher], original copyright [original copyright year].”
 
2. If you are unable to obtain permission from the original copyright holder to publish these figures under the CC BY 4.0 license or if the copyright holder’s requirements are incompatible with the CC BY 4.0 license, please either i) remove the figure or ii) supply a replacement figure that complies with the CC BY 4.0 license. Please check copyright information on all replacement figures and update the figure caption with source information. If applicable, please specify in the figure caption text when a figure is similar but not identical to the original image and is therefore for illustrative purposes only.
The following resources for replacing copyrighted map figures may be helpful:
 
USGS National Map Viewer (public domain): <http://viewer.nationalmap.gov/viewer/>
The Gateway to Astronaut Photography of Earth (public domain): <http://eol.jsc.nasa.gov/sseop/clickmap/>
Maps at the CIA (public domain): <https://www.cia.gov/library/publications/the-world-factbook/index.html> and <https://www.cia.gov/library/publications/cia-maps-publications/index.html>
NASA Earth Observatory (public domain): <http://earthobservatory.nasa.gov/>
Landsat: <http://landsat.visibleearth.nasa.gov/>
USGS EROS (Earth Resources Observatory and Science (EROS) Center) (public domain): [http://eros.usgs.gov/#](http://eros.usgs.gov/)
Natural Earth (public domain): <http://www.naturalearthdata.com/>

Response: All figures in this manuscript were generated by the authors using R and contain no copyrighted materials.

4. Please provide additional details regarding participant consent. In the ethics statement in the Methods and online submission information, please ensure that you have specified (1) whether consent was informed and (2) what type you obtained (for instance, written or verbal, and if verbal, how it was documented and witnessed). If your study included minors, state whether you obtained consent from parents or guardians. If the need for consent was waived by the ethics committee, please include this information.

If you are reporting a retrospective study of medical records or archived samples, please ensure that you have discussed whether all data were fully anonymized before you accessed them and/or whether the IRB or ethics committee waived the requirement for informed consent. If patients provided informed written consent to have data from their medical records used in research, please include this information.

Please review your reference list to ensure that it is complete and correct. If you have cited papers that have been retracted, please include the rationale for doing so in the manuscript text, or remove these references and replace them with relevant current references. Any changes to the reference list should be mentioned in the rebuttal letter that accompanies your revised manuscript. If you need to cite a retracted article, indicate the article’s retracted status in the References list and also include a citation and full reference for the retraction notice.

Response: Thank you for noting this. We have added the following language to the methods section just before the description of ethical approval: “Written informed consent was obtained for all adult participants and captured electronically. Parental/guardian written consent for minors aged 14 year and under, and written assent from children aged 10-14 years, was captured electronically.”

Additional Editor Comments (if provided):

Dear Authors,

The following comments were raised by the reviewers. Kindly respond to these comments.

**REVIEWER 1**

Great efforts put in to utilized population-based HIV survey for evidence or targeted interventions to prevent new HIV infection and HIV control in a resource-limited nation is good innovation.
The STATISTICAL METHODS is robust and results unambiguous for policy guidance in HIV/AIDS programming. However, there are many claims that ensemble models used in your statistical analysis could be shrouded with generalization errors, how did you overcome overfitting models in your analysis?
Data access was adequate and useful in buttressing the credibility of the manuscript.

Response: Thank you to the reviewer for their thorough review and insightful comments. We agree with the reviewer that there can be concerns about over-fitting with the type of ensemble model that we utilized for HIV prevalence (note that the models for ART and VLS do not include this ensemble modeling component). Following Bhatt and colleagues (citation 25 in the main text), as well as previous applications of a similar approach to modeling HIV prevalence (citation 1 in the main text), we mitigate the risk of over-fitting via a cross-validation approach. Specifically, the observations in the full dataset are divided into five groups and each of the sub-models is fit five times, excluding each group of data in turn. Out-of-sample predictions are then produced for the excluded data for each model, which leads to a full set of out-of-sample predictions for each sub-model. These out-of-sample predictions from the sub-models are then used in fitting the geo-statistical model. The sub-models are also fit on the full dataset, and this is used to generate a full set of in-sample predictions for each sub-model. These in-sample predictions, plus the fitted geo-statistical model, are then used to produce the final estimates. By leveraging out-of-sample predictions throughout the model fitting process, we prioritize models that generalize well, rather than models that potentially over-fit the observed data.

**REVIEWER 2**

The strength of the study lies on the used household surveys and GPS to analyze data for HIV prevalence, ART and VLS.
This is a better model than the State based approaches used presently in the country for policy making. However, the challenge is that the use of these databases have underlying assumptions, as stated by the authors as limitations to the study, use of household data may not correctly capture high risk groups, vulnerable populations and marginalized communities. Also, the use of GPS may incorrectly capture coordinates for all the locations. These limitations will hamper the reliability of the results.
Suggestion: There are other software used by medical geographers and geospatial analysts that could be used alongside the data used in this study to further enhance the accuracy of the results and also eliminate the bias further. It is not out of place if the authors engage these specialists in order to further improve the quality of the work.

Response: Thank you to the reviewer for their careful review and thoughtful comments. We agree with the reviewer that a more detailed spatial analysis like this one reveals important additional information and enables insights beyond what is afforded by state-level analysis alone. We also agree that there are a number of challenges inherent in the analysis of household survey data in general and particularly in the context of geospatial analysis, including concerns related to survey coverage and representativeness and the errors introduced by displacing GPS coordinates in order to protect respondents’ confidentiality, and so we explicitly acknowledge these concerns in the section on limitations in the discussion. While there are a variety of approaches that have been employed for this type of detailed spatial analysis, we believe that the particular approach used in this manuscript is both appropriate given the research questions and comparable to methodologies regularly utilized by other experts in the field. We also note that a number of authors of this manuscript, including those most closely involved in the development of the statistical models, could reasonably be considered specialists in geospatial analysis.

Specific suggestions: Replace "prevalence of ART" with "ART coverage"
Replace "prevalence of VL suppression" with "virus suppression rate"

Response: The revised manuscript includes the reviewers suggested changes.
